# Physiological Fitness and the Pathophysiology of Chronic Lymphocytic Leukemia (CLL)

**DOI:** 10.3390/cells10051165

**Published:** 2021-05-11

**Authors:** Andrea Sitlinger, Michael A. Deal, Erwin Garcia, Dana K. Thompson, Tiffany Stewart, Grace A. MacDonald, Nicolas Devos, David Corcoran, Janet S. Staats, Jennifer Enzor, Kent J. Weinhold, Danielle M. Brander, J. Brice Weinberg, David B. Bartlett

**Affiliations:** 1 Hematologic Malignancies and Cellular Therapy, Duke University School of Medicine, Durham, NC 27705, USA; andrea.sitlinger@duke.edu (A.S.); danielle.brander@duke.edu (D.M.B.); 2 Division of Medical Oncology, Duke University School of Medicine, Durham, NC 27710, USA; dealma@mail.uc.edu (M.A.D.); gmaclax@gmail.com (G.A.M.); 3 Duke Molecular Physiology Institute, Duke University School of Medicine, Durham, NC 27701, USA; 4 Laboratory Corporation of America Holdings, Morrisville, NC 27560, USA; garce14@labcorp.com; 5 VA Medical Center, Division of Hematology, Duke University School of Medicine, Durham, NC 27710, USA; dana.thompson@duke.edu (D.K.T.); tiffany.stewart@duke.edu (T.S.); brice@duke.edu (J.B.W.); 6 Duke Center for Genomic and Computational Biology, Duke University, Durham, NC 27705, USA; Nicolas.devos@duke.edu (N.D.); David.corcoran@duke.edu (D.C.); 7 Division of Surgical Sciences, Duke University School of Medicine, Durham, NC 27707, USA; janet.staats@duke.edu (J.S.S.); jennifer.enzor@duke.edu (J.E.); kent.weinhold@duke.edu (K.J.W.)

**Keywords:** aging, physical fitness, NMR lipoprotein and inflammation, NGS exosomal miRNA, NK cell phenotype

## Abstract

Chronic lymphocytic leukemia (CLL) is associated with physical dysfunction and low overall fitness that predicts poor survival following the commencement of treatment. However, it remains unknown whether higher fitness provides antioncogenic effects. We identified ten fit (CLL-FIT) and ten less fit (CLL-UNFIT) treatment-naïve CLL patients from 144 patients who completed a set of physical fitness and performance tests. Patient plasma was used to determine its effects on an *in vitro* 5-day growth/viability of three B-cell cell lines (OSU-CLL, Daudi, and Farage). Plasma exosomal miRNA profiles, circulating lipids, lipoproteins, inflammation levels, and immune cell phenotypes were also assessed. CLL-FIT was associated with fewer viable OSU-CLL cells at Day 1 (*p* = 0.003), Day 4 (*p* = 0.001), and Day 5 (*p* = 0.009). No differences between the groups were observed for Daudi and Farage cells. Of 455 distinct exosomal miRNAs identified, 32 miRNAs were significantly different between the groups. Of these, 14 miRNAs had ≤−1 or ≥1 log2 fold differences. CLL-FIT patients had five exosomal miRNAs with lower expression and nine miRNAs with higher expression. CLL-FIT patients had higher HDL cholesterol, lower inflammation, and lower levels of triglyceride components (all *p* < 0.05). CLL-FIT patients had lower frequencies of low-differentiated NKG2+/CD158a/b^neg^ (*p* = 0.015 and *p* = 0.014) and higher frequencies of NKG2A^neg^/CD158b+ mature NK cells (*p* = 0.047). The absolute number of lymphocytes, including CD19+/CD5+ CLL-cells, was similar between the groups (*p* = 0.359). Higher physical fitness in CLL patients is associated with altered CLL-like cell line growth *in vitro* and with altered circulating and cellular factors indicative of better immune functions and tumor control.

## 1. Introduction

Chronic lymphocytic leukemia (CLL) is the most prevalent adult leukemia in the USA [1,2], with a median age at diagnosis of approximately 70 years [3,4]. The median overall survival is approximately 10 years, with durations ranging from months to decades [5]. The presentation of CLL is diverse, and patients have a shorter life expectancy than age-matched healthy populations [6]. CLL increases the risk of secondary malignancies and autoimmune diseases, and infections are the leading cause of death [7,8,9]. At present, there is no survival benefit from immediate or early therapy prior to the established treatment, and most patients have a period of observation before therapy initiation [10,11]. During the treatment-naïve period, patients with CLL can have low overall fitness and physical dysfunction, both of which predict poor survival following the commencement of treatment [12]. To date, we are aware of no studies that have assessed the role physical fitness has on the underlying pathophysiological factors of CLL [13]. 

Higher physical fitness and physical activity levels for lymphoma patients are associated with improve ments in therapy-related side effects, physical functioning, and quality of life [13,14,15]. Although the underlying mechanisms are not fully understood, these effects can be partly explained by systemic changes or differences in the host pathways, including metabolism, inflammation, and immune function, that promote a less carcinogenic milieu [16]. In healthy adults, a higher physical fitness and following a single bout of exercise can result in the reduced *in vitro* growth of breast, prostate, and colorectal cancer cell lines using autologous serum [17,18,19,20]. In these studies, exercise was associated with transient inflammatory changes, suggesting complex interactions of inflammatory mediators on cell line growth in vitro. Since elevated levels of pro-inflammatory mediators are hallmarks of certain cancers, including CLL [21,22,23], other physical fitness host pathways are also likely to be influencing cancer behaviors in vivo [24]. 

The primary aim of our current study was to evaluate the roles of high and low physical fitness plasma from treatment-naïve CLL patients on the in vitro growth/survival of a CLL-like cell line (OSU-CLL) [25]. The secondary aim was to identify the similarities and differences in (a) circulating factors and (b) mononuclear cell phenotypes associated with CLL pathology. We hypothesized that plasma from high physically fit CLL patients would reduce the growth of OSU-CLL cells *in vitro*. 

## 2. Materials and Methods

### 2.1. Patient Characteristics

Ten physically fit (CLL-FIT) and 10 physically less fit (CLL-UNFIT) treatment-naïve CLL patients matched by age (mean ± SD: 67.8 ± 10.8 y, range: 51–86 y) and sex (5M/5F in each group) were assessed for this study. Study participants were identified from 144 CLL patients assessed for physical fitness and function during their regular clinical visits at the Duke Cancer Center between July 2017 and March 2018. Of these patients, 69 were confirmed treatment = naïve and Rai Stage 0 to 1. All participants gave written informed consent, and the study was approved by the Duke University Medical Center Institutional Review Board.

### 2.2. Clinical Characteristics

Clinical indices were obtained from the patients’ medical records. These included the CLL-IPI scores, calculated as previously described [26], disease duration, cytogenetics (i.e., standard karyotype and fluorescent in situ hybridization for CLL), IGHV mutation status, and CD38 expression. Plasma levels of soluble CD20 (sCD20) and intercellular adhesion molecule 1 (ICAM-1) were determined in duplicate using a human sandwich immunoassay according to the manufacturer’s instructions (Meso Scale Discovery, Rockville, MD, USA). β2-microglobulin (B2M) was determined in duplicate using a commercially available ELISA (R&D Systems, Minneapolis, MN, USA). The lower limits of detection (LLOD) were sCD20 (30.5 pg/mL), ICAM-1 (2.60 ng/mL), and B2M (0.132 µg/mL). All samples had concentrations greater than the LLOD with the exception of sCD20, with 81% of samples above the LLOD. Complete blood counts (CBCs) and differentials were clinically assessed at the Duke Clinical Laboratory using an automated hematology analyzer (Sysmex, Lincolnshire, IL, USA).

### 2.3. Physical Performance and Fitness

Patients completed a short battery of standardized physical performance tests. These included the 6-min walk test (6MWT), the short physical performance battery (SPPB), timed-up and go (TUG), and grip strength. Following the completion of physical testing, patients completed two physical activity questionnaires—the Incidental and Planned Activity Questionnaire (IPAQ) and the Stanford Brief Activity Survey (SBAS). We measured height and weight before testing, and blood pressure and resting heart rate following 10 min of seated rest. The eVO_2peak_ (mL/kg/min) was calculated using a validated equation that incorporates the 6MWT distance, resting heart rate, weight, sex, and age [27]. We stratified groups using eVO_2peak_ matched for age (±3 y) and gender.

### 2.4. Blood Sampling

Approximately 20 mL of blood was collected into vacutainers containing either EDTA or heparin as an anticoagulant. Blood was centrifuged at 3000× rpm for 10 min at 4 °C, and 3 to 4 mL of plasma was aliquoted and immediately frozen at −80 °C. From the remaining blood, mononuclear cells (PBMCs) were isolated using Ficoll-Hypaque (Cytavia, Marlborough, MA, USA) density centrifugation and stored in aliquots of 10 × 10^6^ cells/mL in 90% fetal bovine serum (FBS) plus 10% DMSO in the vapor phase of liquid nitrogen [28].

### 2.5. Autologous Plasma Incubation with Cell Lines

We acquired the OSU-CLL cell line, generated by EBV transformation, from the Byrd Lab at Ohio State University [25]. This cell line is characterized by CD5 positivity, mutated IGHV, trisomy 12 and trisomy 19, a noncomplex karyotype, and wild-type p53 expression. Importantly, it is stable under extended periods of culture. In addition to the OSU-CLL line, we used two other B-cell lymphoma cell lines to assess the potential similarities between the cell growth and plasma characteristics. Specifically, we used the Daudi (ATCC^®^ CCL-213) and Farage (ATCC^®^ CRL-2630) cell lines. The Daudi line is an EBV-transformed Burkitt’s lymphoma B-lymphocyte, while the Farage is an EBV-transformed mature B-cell line from a patient with diffuse large cell non-Hodgkin’s lymphoma. The Daudi line is CD19+, CD5^neg^, and β2-microglobulin-negative, and the Farage line is CD19+, CD20+, CD5^neg^, and HLA-DR+. Cells were thawed and grown for 7 days in complete medium (RPMI + 56 U/mL penicillin + 56 µg/ml streptomycin + 2 mM L-glutamine) + 10% fetal bovine serum (FBS). Cells were then washed and resuspended at 5 × 10^5^ cells/mL in complete media + 10% FBS (control) or had the FBS replaced with 10% autologous plasma. To determine whether the differences between the controls (serum) and autologous plasma might be affected by anticoagulant agents, we also incubated the serum (collected at the same time for a different study) from four patients with the OSU-CLL line. Correlations between the autologous serum and plasma were *r* = 0.85 (data not shown). At baseline and every 24 hours for 5 days, cell density was measured in duplicate by both hemocytometer counting and the cell-counting feature of the Attune NxT flow cytometer (Thermo Fisher, Waltham, MA, USA). Live, viable cells were quantified as Annexin^neg^/PI^neg^, early apoptotic as Annexin^pos^/PI^neg^, and late apoptotic/necrotic as Annexin^pos^/PI^pos^ using the manufacturer’s guidelines (BD Biosciences, San Jose, CA, USA) with the Attune NxT flow cytometer. 

### 2.6. Exosomal miRNA

Plasma (500 µL) was briefly thawed at 37 °C and filtered to exclude particles >0.8 µm for the isolation of exosomal RNA using the Qiagen exoRNeasy mini kit (Qiagen, Hilden, Germany). We then purified the exosomes before RNA extraction was completed, using Qiagen miRNeasy chloroform-based technology. The RNA quantity and quality were quantified on a NanoDrop 2000 spectrophotometer (Thermo Fisher, Waltham, MA, USA). For sequencing, 5 µL of each exosomal RNA extract was used to generate a miRNA library using principals and methods similar to those previously described [29,30,31]. The libraries were generated using the QIAseq miRNA Library kit (Qiagen). This kit uses modified adapters that efficiently ligate to microRNAs generated by Dicer processing. It also uses 12-bp Unique Molecular Indices (UMIs) to tag each miRNA at an early stage, eliminating the PCR and sequencing bias. During library prep, the libraries were indexed using a single 6-bp indexing approach, allowing for multiple libraries to be pooled and sequenced on the same sequencing flow cell of an Illumina sequencing platform. Before pooling and sequencing, we assessed the fragment length distribution and overall library quality on an Agilent Fragment Analyzer instrument (Agilent Technologies, Santa Clara, CA, USA). The concentrations of each library were assessed using a Qubit fluorometer (Thermo Fisher, Waltham, MA, USA). We then pooled 20 libraries in an equimolar ratio and sequenced them on a NextSeq High Output flow cell. To sequence the 12-bp UMIs that were used to tag each miRNA, the sequencing was completed at a 75-bp single read. Approximately sixteen million reads were generated for each sample, and a total of 455 distinct miRNAs were identified.

#### miRNA Analyses

smRNA-seq data was processed using the TrimGalore toolkit [32] that employs Cutadapt [33] to trim low-quality bases and Illumina sequencing adapters from the 3’ end of the reads. Only reads that were 18 to 28 nt in length after trimming were kept for further analysis. Reads were mapped to the hg19 version of the human genome using the Bowtie alignment tool [34]. Reads were kept for subsequent analysis if they mapped to no more than 13 genomic locations. Gene counts were compiled using custom scripts that compare mapped read coordinates to the miRBase [35] microRNA database. Reads that matched the coordinates of the known mature microRNAs were kept if they perfectly matched the coordinates of the miRNA seed while not varying by more than 2 nt on the 3’ end of the mature miRNA. Only mature miRNAs that had at least 10 reads in any given sample were used in the subsequent analysis. Normalization and differential expression were carried out using the DESeq2 [36] Bioconductor [37] package from the *R* statistical programming environment. Normalization was performed using the ‘*poscounts*’ approach to eliminate the systematic differences across the samples. 

### 2.7. Flow Cytometry

PBMCs were partly thawed in a 37 °C water bath, then completely thawed using the dropwise method and washed in a 37 °C thaw buffer (RPMI + 10% FBS + 1% penn/strep + 1% L-Glutamine + 25-U/µL Benzonase). Cells were counted and resuspended at 10 × 10^6^ cells/mL in Dulbecco’s PBS (DPBS), and 100 µL were aliquoted into FACS tubes. Cells were first stained with 0.1 µL of the viability dye Zombie Aqua (BioLegend, San Diego, Ca, USA) before blocking Fc receptors (Human TruStain FcX, BioLegend, San Diego, CA, USA), followed by complete combinations of the following antibodies. The NK cell tubes contained 0.63 µg/mL CD3 BUV395 (Clone SK7; BD Biosciences, San Jose, CA, USA), 0.16 µg/mL CD56 BB700 (Clone NCAM16.2; BD Biosciences, San Jose, CA, USA), 0.25 µL NKG2A PE (Clone REA110; Miltyeni Biotec, Gaithersburg, MD, USA), 2.5 L NKG2C PE-Vio770 (Clone REA205; Miltyeni Biotec, Gaithersburg, MD, USA), 10 µg/mL NKG2D BB515 (Clone 1D11; BD Biosciences, San Jose, CA, USA), 5 µg/mL CD244 BV421 (Clone 2–69; BD Biosciences, San Jose, CA, USA), 5 µL CD158a APC-Vio770 (Clone REA284; Miltyeni Biotec, Gaithersburg, MD, USA), and 5 µg/mL CD158b APC (Clone DX27; BioLegend, San Diego, CA, USA). Monocyte tubes contained 1.25 µg/mL CD14 BUV805 (Clone M5E2; BD Biosciences, San Jose, CA, USA) and 1.25 µg/mL CD16 BUV395 (Clone 3G8; BD Biosciences, San Jose, CA, USA). T-cell and B-cell tubes contained 1 µg/mL CD3 Pacific Blue (Clone UCHT1; BD Biosciences, San Jose, CA, USA), 1 µg/mL CD4 PE (Clone OKT4; BioLegend, San Diego, CA, USA), 10-µg/mL CD8 FITC (Clone OKT8; Thermo Fisher, Waltham, MA, USA), 1.5 µg/mL CD19 APC-Cy7 (Clone HIB19; BioLegend, San Diego, CA, USA), and 3 µg/mL CD5 APC (Clone UCHT2; BioLegend, San Diego, CA, USA). We titrated all the antibodies prior to assessing the samples and used single color and flow minus one (FMOs) tubes for compensation. Cells were incubated for 30 min on ice in the dark before being fixed with 1% paraformaldehyde (Sigma Aldrich, St. Louis, MO, USA). Cells were analyzed on either a BD LSR Fortessa (NK cell and monocytes) equipped with 4 lasers or a BD FACS Canto II (T cells and B cells) equipped with 3 lasers. All analyses were completed after acquisition using FCS Express v6 (DeNovo Software, Pasadena, CA, USA).

### 2.8. Nuclear Magnetic Resonance (NMR) Spectroscopy

Plasma (600 µL) was analyzed by NMR at LabCorp (Morrisville, NC, USA) as a single batch. NMR spectra were acquired on a Vantera^®^ Clinical Analyzer, as previously described [38]. The concentration of GlycA, a marker of systemic inflammation [39,40], was calculated from NMR signal amplitudes of highly mobile protons of *N*-acetylglucosamine residues located on the carbohydrate side chains of circulating acute-phase proteins (e.g., α1-acid glycoprotein, haptoglobin, α1-antitrypsin, α1-antichymotrypsin, and transferrin). Concentrations of lipids, lipoprotein particles, apolipoproteins, and particle sizes were measured using an advanced proprietary deconvolution algorithm (LP4) that provides a better resolution of subclasses compared to previous algorithms [38,41,42]. We calculated the Lipoprotein Insulin Resistance Index (LP-IR) from the NMR-measured lipoprotein particle sizes (TRL, LDL, and HDL) and particle concentrations (very large TRL + large TRL, small LDL, and large HDL). LP-IR scores ranged from 0 (least insulin resistant) to 100 (most insulin resistant) [38,41]. Valine, leucine, and isoleucine (BCAAs) and their sum (total BCAA) were quantified as previously described [43]. Glucose, glycine, and alanine were measured using the LP4 algorithm.

### 2.9. Statistical Analyses

We conducted patient characteristics, NMR data, and immune data analyses using SPSS version 23.0 (IBM, Armonk, NY, USA). Normality was assessed using the Kolmogorov–Smirnov analysis. For the variables violating normality, we used nonparametric analyses. Comparisons of variables were completed using independent *t*-tests and Mann–Whitney *U* tests, and chi-square analyses were used for categorical variables. Spearman correlations were conducted between variables as measures of associations. For the analyses of cell line incubations with autologous plasma, a repeated linear mixed model was used to model both the numeric and percentage changes in cell growth. The model included the main effects and interaction effects for time (Days 0, 1, 2, 3, 4, and 5) and group (CLL-FIT and CLL-UNFIT). Contrasts were used a priori to determine the overall effects for time within each group, where significant effects for time occurred and differences between groups at each time point. The results are presented with standard deviations (SD) and 95% confidence intervals (CI) and the effect sizes calculated as Cohen’s D (*d*). Statistical significance was accepted as *p* ≤ 0.05. 

## 3. Results

### 3.1. Group Demographics, Clinical Measures, Physical Fitness, and Function

Groups were clinically similar for staging, disease duration, and cytogenetics (all *p* > 0.1) (Table 1). CLL-UNFIT patients were heavier (*p* = 0.049) and had a higher BMI (*p* = 0.013). CLL-FIT had higher cardiorespiratory fitness based on eVO_2peak_ (*p* < 0.001), which was characterized by CLL-FIT completing a 20% greater 6MWT distance (*p* = 0.027) and 18% better on the SPPB (*p* = 0.012). Both groups had similar grip strengths, TUG times, and similar self-reported exposures to the physical activity levels (all *p* > 0.05). CLL-UNFIT had higher neutrophil counts (*p* = 0.046), while the lymphocyte (*p* = 0.517) and monocyte counts (*p* = 0.694) were similar in both groups. No differences were observed between groups for the absolute numbers of the CD19+/CD5+ CLL B cells (*p* = 0.314), CD4+ (*p* = 0.169), or CD8+ (*p* = 0.088) T cells. Similarly, no differences were observed for CD14+/CD16^neg^ (*p* = 0.861), CD14+/CD16+ (*p* = 0.635), or CD14+/CD16++ (*p* = 0.598) monocytes (data not shown).

### 3.2. Cell Line Growth with Autologous Plasma

**Figure 1:**
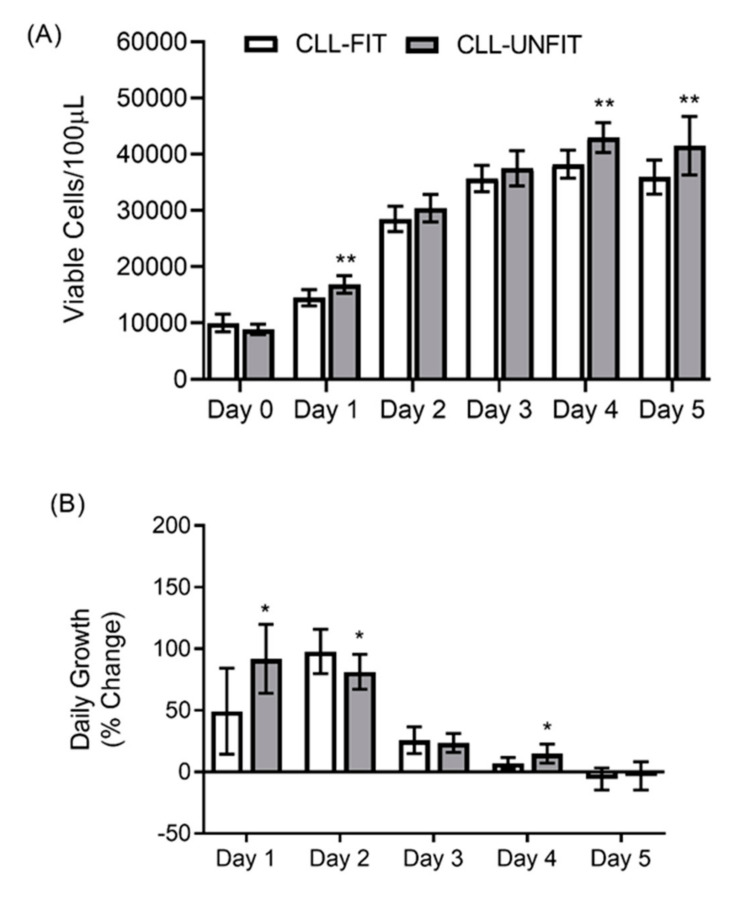
5-day cell growth in the presence of complete media + 10% autologous plasma from CLL-FIT and CLL-UNFIT. (**A**) OSU-CLL viable cell counts each day. (**B**) OSU-CLL viable percentage change from the previous day. * *p* < 0.05 and ** *p* < 0.01 different than CLL-FIT at that corresponding time point. Data are mean ± SD.

Cell growth was quantified by both the average live cell count per well from duplicate wells and the percentage change of the cell counts over time in the culture (Figure 1). The results are representative from ten CLL-FIT and nine CLL-UNFIT patients. There was a significant main effect for time (F (5, 85) = 615.0; *p* < 0.001; η^2^ = 0.973) and a group x time interaction (F (5, 85) = 5.17; *p* < 0.001; η^2^ = 0.233) for the viable OSU-CLL cell numbers (Figure 1A). At Day 1 (*p* = 0.003), Day 4 (*p* = 0.001), and Day 5 (*p* = 0.009), CLL-UNFIT had 16%, 12%, and 15.5% more cells than CLL-FIT did, respectively. Similarly, there was a significant main effect for time (F (4, 68) = 88.1; *p* < 0.001; η^2^ = 0.838) and a group x time interaction (F (4, 68) = 6.57; *p* < 0.001; η^2^ = 0.279) for a viable OSU-CLL percentage change from the previous day (Figure 1B). Compared to CLL-FIT, at Day 1 (*p* = 0.010) and Day 4 (*p* = 0.016), CLL-UNFIT had a 42.5% and 7.7% greater increase in the previous day’s cell numbers but a 16.5% lower increase in the previous cell numbers at Day 2 (*p* = 0.042). No group differences were observed for the Daudi or Farage cell lines (data not shown). 

### 3.3. Exosomal microRNA Profiles

**Figure 2:**
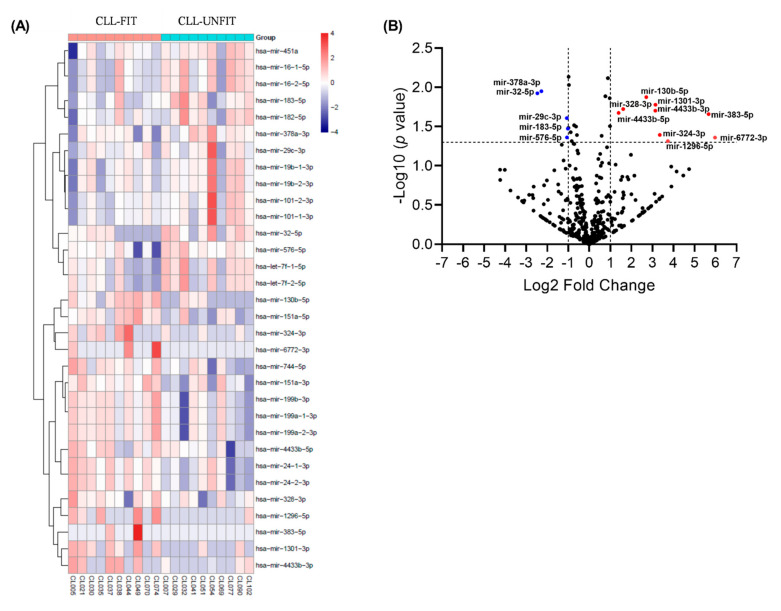
Differential expression of plasma exosomal miRNAs between CLL-FIT and CLL-UNFIT. (**A**) Heat map with a dendrogram illustrating the correlation distance with complete linkage hierarchal clustering for the 32-exosomal miRNAs differentially expressed when comparing CLL-FIT to CLL-UNFIT. Values represent the z-score normalized miRNA counts per million reads. Colors represent the level of miRNA expression, with dark red being the highest expression and dark blue the lowest expression. (**B**) Volcano plot of differentially expressed miRNAs in CLL-FIT vs. CLL-UNFIT. The *p*-value in the −log10 scale is plotted against the log2 fold change for each miRNA, with each circle denoting an individual miRNA. The miRNAs with Wald Test *p*-values ≤ 0.05 and log2 fold changes ≥1 or ≤−1 are represented by red circles (higher expression in CLL-FIT) or blue circles (higher expression in CLL-UNFIT). Nine exosomal miRNAs were higher and five were lower in the CLL-FIT compared to CLL-UNFIT group.

RNAseq revealed a total of 455 distinct exosomal miRNA profiles isolated from patient plasma samples (Supplementary Table S1). To assess the similarities and differences in exosomal miRNA profiles between CLL-FIT and CLL-UNFIT patients, we employed a hierarchical clustering method. As shown in Figure 2A, exosomal miRNA clusters in CLL-FIT vs. CLL-UNFIT were characterized by 32 exosomal miRNAs, having a Wald Test *p*-value of ≤0.05 (Supplementary Table S2). Of these, 14 miRNA profiles had ≤−1 or ≥1 log2 fold differences between the groups (Figure 2B). Relative to CLL-UNFIT, CLL-FIT had five miRNAs (miR-378a-3p, miR-32-5p, miR-29c-3p, miR-183-5p, and miR-576-5p) with lower levels (blue dots) and nine miRNAs (miR-130b-5p, miR-1301-3p, miR-4433b-3p, miR-383-5p, miR-328-3p, miR-4433b-5p, miR-324-3p, miR-6772-3p, and miR-1296-5p) with higher levels (red dots). Using miRNA target gene prediction software (miRDB: http://mirdb.org/index.html, accessed on 8 July 2020), we determined which target genes relevant to CLL would be affected by the differential expression of exosomal miRNAs. There was a pattern for the miRNAs higher in CLL-UNFIT exosomes to target NOTCH, BCL2, and cyclin signaling.

### 3.4. NK Cell Immunophenotype

**Figure 3:**
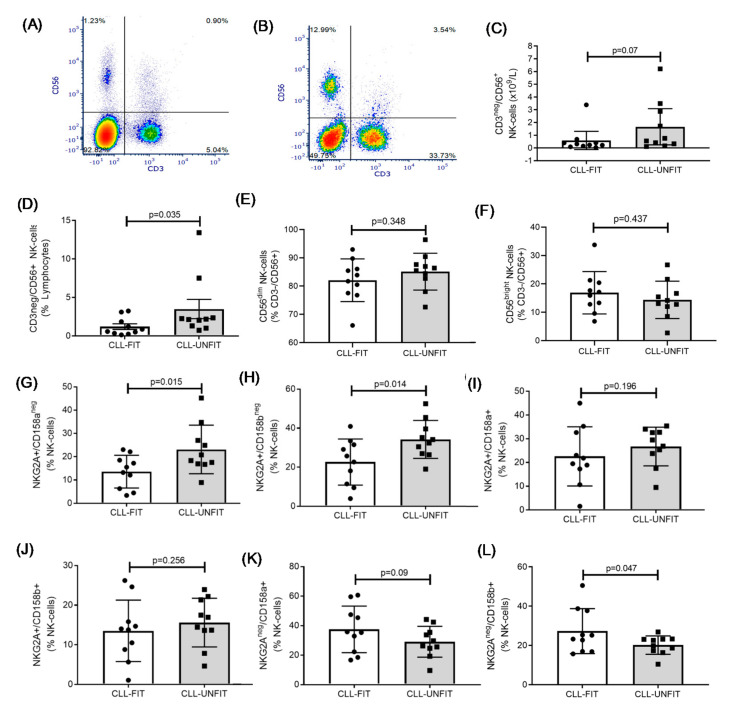
NK cell similarities and differences between CLL-FIT and CLL-UNFIT. Representative flow cytometry plots of CD3 vs. CD56 lymphocytes for (**A**) CLL-FIT and (**B**) CLL-UNFIT. (**C**) Absolute numbers of CD3^neg^/CD56+ NK cells. (**D**) Percentage of lymphocytes that are CD3^neg^/CD56+ NK cells. (**E**) Frequency of CD56^dim^ NK cells within the NK cell population. (**F**) Frequency of CD56^bright^ NK cells within the NK cell population. (**G**) Frequency of least-differentiated NKG2A+/CD158a^neg^ NK cells. (**H**) Frequency of least-differentiated NKG2A+/CD158b^neg^ NK cells. (**I**) Frequency of medium-differentiated NKG2A+/CD158a+ NK cells. (**J**) Frequency of medium-differentiated NKG2A+/CD158b+ NK cells. (**K**) Frequency of most-differentiated NKG2A^neg^/CD158a+ NK cells. (**L**) Frequency of most-differentiated NKG2A^neg^/CD158b+ NK cells. Data are the mean and 95% CI.

Figure 3A (CLL-FIT) and Figure 3B (CLL-UNFIT) show representative flow cytometry plots for the frequencies of CD3^neg^/CD56+ NK cells on total lymphocytes. CLL-FIT patients had a trend for lower absolute numbers of NK cells (Figure 3C: *p* = 0.07) and lower frequencies of CD3^neg^/CD56+ NK cells (Figure 3D: *p* = 0.035). Groups were similar for the frequencies of CD56^dim^ (Figure 3E: *p* = 0.348) and CD56^bright^ (Figure 3F: *p* = 0.437) NK cells. CLL-FIT had a lower frequency of low-differentiated NK cells characterized by NKG2A+/CD158a^neg^ (Figure 3G: *p* = 0.015) and NKGA2A+/CD158b^neg^ (Figure 3H: *p* = 0.014). We noted no differences for medium-differentiated NK cells characterized by NKG2A+/CD158a+ (Figure 3I: *p* = 0.196) or NKG2A+/CD158b+ (Figure 3J: *p* = 0.256). CLL-FIT had a higher frequency of terminally differentiated NK cells characterized by NKG2A^neg^/CD158b+ (Figure 3L: *p* = 0.047) but not NKG2A^neg^/CD158a+ (Figure 3K: *p* = 0.09). Of the NK cell surface marker expression (MFI), only CD158a was higher in CLL-FIT (257 ± 32 vs. 229 ± 24, *p* = 0.046), with no differences for CD158b (257 ± 113 vs. 217 ± 39, *p* = 0.301), NKG2A (769 ± 425 vs. 762 ± 134, *p* = 0.960), NKG2C (299 ± 58 vs. 264 ± 22, *p* = 0.093), NKG2D (1293 ± 220 vs. 1331 ± 133, *p* = 0.651), or CD244 (141 ± 48 vs. 120 ± 5, *p* = 0.190), data not shown.

### 3.5. NMR Measured Lipids, Lipoproteins (LipoProfile^®^), and Inflammatory Profiles

Table 2 shows the group differences for the pertinent NMR measures. CLL-FIT had lower GlycA concentrations (*p* = 0.014) and a lower lipoprotein-derived insulin resistance score (LP-IR: *p* = 0.007). CLL-FIT had lower concentrations of total triglycerides (*p* = 0.025) and triglyceride-rich lipoproteins (TRLs; *p* = 0.016). CLL-FIT had lower total levels of TRL particles (TRLP: *p* = 0.046), large TRLP (*p* = 0.011), and very small TRLP (*p* = 0.044). CLL-FIT had smaller mean TRL particle sizes (*p* = 0.027). CLL-FIT had lower levels of TRL cholesterol (*p* = 0.034), higher HDL cholesterol (*p* = 0.040), and higher concentrations of large HDL particles (*p* = 0.043).

### 3.6. Correlations between miRNAs, Immune Cells, and Lipids

CLL-UNFIT patients had higher expressions of miR-29c that were associated with higher WBC (*r* = 0.558, *p* = 0.011) and lymphocyte counts (*r* = 0.555, *p* = 0.011). CLL-FIT patients had higher expressions of miR130b-5p that were associated with lower absolute counts of CD56^bright^ (*r* = −0.493, *p* = 0.027) and a trend for CD56^dim^ (*r* = −0.434, *p* = 0.056) NK cells. Additionally, a higher expression of miR-130b was associated with lower frequencies of low-differentiated NKG2A+/CD158a^neg^ (*r* = −0.544, *p* = 0.013) and highly differentiated mature NKG2A+/CD158b^neg^ NK cells (*r* = −0.461, *p* = 0.041). Higher expressions of miR-130b were associated with higher levels of large HDL particles (*r* = 0.489, *p* = 0.029), lower insulin resistance (*r* = −0.498, *p* = 0.025), lower large TRLP levels (*r* = −0.497, *p* = *−*0.026), and smaller TRL lipoprotein sizes (*r* = −0.451, *p* = 0.046). Finally, higher expressions of miR-4433b-3p were associated with higher frequencies of highly differentiated NKG2A^neg^/CD158a+ (*r* = 0.459, *p* = 0.042) and a trend for higher NKG2A^neg^/CD158b+ NK cells (*r* = 0.390, *p* = 0.089).

## 4. Discussion

For the first time, to our knowledge, we have examined the role of physical fitness on *in vitro* malignant cell line growth, circulating factors, and immunophenotype in older adults with treatment-naïve chronic lymphocytic leukemia. The incubation of patient-derived plasma with leukemia/lymphoma cell lines was associated with the differential growth patterns of a CLL-like cell line (OSU-CLL), suggesting that components in the plasma are specific for the growth of CLL-like cells. In an attempt to understand the lower OSU-CLL growth by CLL-FIT plasma, we assessed soluble mediators with known roles in worsening CLL pathophysiology. Specifically, we assessed chronic inflammation [21], exosomal miRNAs [44,45], lipids/lipoproteins [46,47], and important immune cell phenotypes [48,49]. We discovered that higher fitness is associated with lower chronic inflammation and identified 32 distinct exosomal miRNA profiles with significantly different expression profiles. We discovered that higher fitness is associated with higher HDL cholesterol, lower triglyceride species (including large and very small triglyceride-rich lipoprotein particle levels), triglyceride-rich lipoprotein cholesterol levels, and smaller triglyceride-rich lipoprotein particle sizes. Finally, higher fitness was associated with a lower frequency of NK cells but a greater frequency of mature NKG2A^neg^/KIR+ NK cells. This cross-sectional analysis suggests that physical fitness may play an important role in the underlying biology of CLL. 

Higher physical fitness is associated with greater exposure to physical activity and exercise. Following a single bout of exercise in healthy humans, sera/plasma reduces the growth of colorectal, breast, and prostate cancer cell lines, suggesting that exercise alters the circulating factors capable of slowing the growth of tumor cells [19,20,50]. During and after each bout of exercise, there are transient increases, then decreases, upon exercise completion of circulating concentrations of pro- and anti-inflammatory cytokines [19,20,50,51,52]. Although the resting sera following exercise training from breast cancer survivors did not alter the growth of cancer cell lines, breast cancer treatment disrupts both immune and inflammatory regulations for extended periods [19]. Here, we observed lower levels of the chronic inflammatory marker GlycA in CLL-FIT patients and slower *in vitro* cell growth. GlycA is robustly modifiable by exercise training and is lower in those with higher cardiorespiratory fitness [53,54]. As such, the role inflammation plays on cancer cell growth is complex, and the inflammatory response to exercise, which promotes higher physical fitness, is equally complex in the cancer setting.

The role of both intracellular and extracellular miRNAs (i.e., cell-free and packaged in extracellular vesicles) in CLL have revealed at least 10–20 miRNAs associated with varying disease characteristics [44,45,55,56,57,58,59]. Exosomes are the smallest known extracellular vesicles (50–100 nm) with distinct biochemical properties that carry cellular components such as proteins, peptides, lipids, mRNAs, and miRNAs [60]. miRNAs are small (~18–28 nt) noncoding RNAs that bind to the specific 3’ UTR of their target mRNA, inducing translational repression, mRNA decapping, and deadenylation [61,62]. miRNAs are transferred from cell to cell in abundance by tumor-derived exosomes and considered as potential disease biomarkers [63,64]. Exosomes are constitutively secreted by CLL cells in response to B-cell receptor activation and stress [65]. The pathophysiological role of these exosomal miRNAs include the promotion of CLL cell survival by (a) altering transcription in the CLL cell by removing tumor-suppressor miRNAs, (b) altering functions of cells of the tumor microenvironment to promote tumor progression, and (c) reducing the effector functions of immune cells such as CD8+ T cells and NK cells to promote immune evasion and immune suppression [63,66,67]. 

Thus far, only one study that we are aware of has assessed the differences in the exosomal miRNA expression between sedentary (*N* = 5) and physically fitter (*N* = 5) older men [68]. In the fitter group, Nair and colleagues found that exosomal miR-486-5p, miR-215-5p, and miR-941were higher but that miR-151b was lower compared to that in the control group, none of which we observed significant differences for in our study [68]. Considering our group consisted of 50% women and all had CLL, the study differences are not surprising. However, we add evidence that physical fitness is likely associated with the differential expression of exosomal miRNAs in older adults. As such, we discuss our results in terms of the known tumor promotion and immune suppression strategies of differentially expressed miRNAs. 

Compared to healthy adults, CLL patients have higher exosomal miR-29c levels that are associated with worsening WBC and lymphocyte counts [45,66,69]. In CLL patients with unfavorable prognostic markers, miR-29c is lower inside CLL cells (rather than in exosomes) [70], suggesting CLL cells expel miR-29c to promote their own survival and growth. Here, we show that CLL-UNFIT patients have higher exosomal miR-29c levels and that higher miR-29c is associated with higher WBC and lymphocyte counts. This suggests that CLL-FIT patients’ exosomal miR-29c are more similar to healthy adults. Additionally, we observed that miR-15a and miR-16 were both approximately two-fold higher in CLL-UNFIT patient exosomes. These exosomal miRNAs were two of the first to be identified as regulators of CLL cell survival and are also lower inside CLL cells [71]. miR-29, miR-15a, and miR-16 target and repress the translation of tumor-suppressor and cell cycle proteins, including NOTCH1, BCL2, and cyclin. As such, by selectively removing them from the tumor cell, tumor cells promote their own survival [72,73].

Other tumor-originating miRNAs function by being secreted in exosomes that deliver the contents to NK cells and T cells and suppress their tumor recognition capacity [73,74]. Of these, miR-29c, miR-378, and miR-183 were higher in exosomes in CLL-UNFIT patients. These miRNAs target and repress NK cell and CD8+ T-cell functions, including IFN-γ production, granzyme B formation, and active receptor expression [75,76,77,78,79]. We observed CLL-UNFIT patients had more NK cells with lower levels of active receptors, suggesting that CLL-UNFIT patient tumor cells secrete more exosomal miRNAs that likely target these effector immune cells. Conversely, miR-130b was higher in CLL-FIT patient exosomes and correlated with lower absolute numbers and frequencies of NK cells, a characteristic noted in healthy adults compared to CLL [48,49]. Of the remaining miRNAs with differing exosomal levels, less is known for CLL. Further studies are required to elucidate whether higher physical fitness promote immune surveillance and reduce immune suppression by selectively increasing (i.e., miR-130b) and lowering (i.e., miR-29c, miR-378, and miR-183) exosomal miRNAs. Consequently, understanding the role of physical fitness on exosomal miRNA functions may lead to novel understandings of CLL progression and immune evasion. 

Exercise and/or weight loss in overweight or obese older adults improves their health by lowering lipids and lipoproteins [80]. We show that our CLL-FIT patients had higher levels of HDL cholesterol and lower triglycerides and triglyceride-rich lipoproteins—levels consistent with better physical fitness [81]. That said, dyslipidemia and hypercholesterol emia have complex and not fully understood roles in the pathogenesis of CLL [82,83]. Compared to healthy adults, CLL patient levels of cholesterol, HDL, and LDL are lower and lowered further as the disease stages increase [84,85]. Previous studies have shown that lipids and LDLs are preferentially utilized by CLL cells for cell signaling functions, improve CLL cell survival, and increase proliferation [46]. However, preventing the CLL cell utilization of lipids (e.g., by lipid-lowering medication) confers a longer time to first treatment and improved CLL patient survival [47,86]. As such, it is likely that a portion of the increased CLL-like cell line growth we observed was because of the differences in the plasma lipid concentrations. Interestingly, we also show that higher exosomal miR-130b levels are associated with better lipid profiles. miR-130b is a regulator of lipid homeostasis, targeting lipid oxidation and LDL receptor expression [87,88]. It is plausible that the higher miR-130b found in CLL-FIT patient exosomes is limiting the CLL-like cell growth utilization of lipids and promoting our observed lower growth. Lowering these lipids and lipoproteins by lifestyle changes before CLL cells can utilize them could modulate CLL cell growth differently and warrants further investigation. 

Higher physical fitness and regular physical activity in older age maintains and improves important immune functions associated with reduced risks of infections, cancer, and chronic diseases [89,90,91,92,93]. However, compared to healthy adults, newly diagnosed and treatment-naive CLL is associated with higher absolute numbers of CD4+ and CD8+ T cells and NK cells [48,49]. We show that CLL-FIT patients have lower frequencies of NK cells and trends for lower absolute numbers of NK cells and CD8+ T cells—all similar to previous observations in healthy adults [48,49]. In CLL, NK and T cells are characterized by an increased inhibitory receptor expression (e.g., PD-1, Tim3, and CTLA-4) indicative of an ‘exhausted’ phenotype, contributing to the poor resolution of infections [94]. Further, CLL reduces NK cell tumor cytotoxicity, reduces the expression of activatory NK cell receptors, and increases inhibitory NK cell receptor expression [48,95]. We show in the current study that our CLL-FIT patients have a lower frequency of inhibitory-positive/activatory-negative NK cells (i.e., NKG2A^+^/KIR^neg^) and a higher frequency of fully competent mature inhibitory-negative/activatory-positive NK cells (i.e., NKG2A^neg^/KIR^+^) that are better capable of recognizing and killing tumor cells [96]. Since we did not observe absolute numerical differences in the malignant cells, it may be that CLL NK cells are less responsive to physical fitness. Further studies are required to determine the role of physical fitness on the normal immune cell phenotype and function in CLL.

Our study is not without limitations. Our sample size was small, and as such, limited our interpretation of the data. Larger sample sizes should be assessed to confirm our findings. We used autologous plasma and fetal bovine serum (control) for the tumor cell incubation assays. We did this to ensure the composition of the plasma used for NMR and RNAseq were similar to the cell assays. Although we tried to encourage all patients to perform their best and to exert maximal effort in their physical assessments, we cannot be certain that this was done, but the differences in other functional measures, such as the SPPB and TUG, did not reflect this. As such, we are confident that our groups are representative of the fitter and least-fit CLL patients. We were unable to characterize differences between the fitness levels and higher and lower BMI. Although this would have provided evidence for the role of body composition, we did not have enough participants to do so. We did not explore the comprehensive phenotypes or functions of T cells or B cells. As such, it remains unclear whether physical fitness is associated with distinct subsets of T and B cells that may be critical to CLL progression. Whether the differences in NK cells better reflects tumor control mechanisms is also unclear. There are two important future directions for our research: (a) confirm our findings in a larger cohort with more comprehensive physiological and biological phenotyping, with the inclusion of more functional measures of tumor control, including cultures of autologous malignant B cells, and (b) attempt to change the physical fitness of CLL patients and determine which of our biological measures changes accordingly. 

## 5. Conclusions

Although our patient numbers were relatively small, we observed in treatment-naïve CLL, the CLL-like cell line (OSU-CLL) cultured *in vitro* with plasma from patients with higher physical fitness results in lower growth as compared to cells cultured in plasma from CLL patients with lower physical fitness. Higher fitness is associated with lower inflammation, differentially expressed miRNAs in plasma exosomes, better lipid profiles, and a greater frequency of mature NK cells. The combination of these factors likely reflects the improved health outcomes associated with being physically fit. Importantly, we noted that physical fitness is not associated with worsening CLL outcomes and suggest that being fitter imparts a reduced risk of cardiovascular disease (i.e., lower GlycA inflammation) and, potentially, less secondary malignancies and infections (i.e., higher frequency of mature NK cells).

## Figures and Tables

**Table 1:** Demographics and clinical characteristics.

	CLL-FIT (*N* = 10)	CLL-UNFIT (*N* = 10)	*p*-Value	95% CI	Effect Size (*d*)
**Demographics**					
Age (years)	65.8 ± 11.1	69.8 ± 10.8	0.426	−14.3, 6.3	0.37
Sex (M/F) ^1^	5/5	5/5	1.000		0.00
Height (cm)	167.8 ± 17.0	170.8 ± 11.9	0.648	−16.8, 10.7	0.20
Weight (kg)	66.2 ± 16.8	85.8 ± 24.1	**0.049**	−39.1, −0.1	0.94
BMI (kg/m^2^)	23 ± 2.9	29 ± 6.2	**0.013**	−10.5, −1.4	1.24
Resting Heart Rate (bpm)	68 ± 12	72 ± 12	0.438	−15.3, 6.9	0.33
**Clinical Characteristics**					
CLL-IPI Score	2.4 ± 2.2	1.3 ± 1.1	0.175	−0.53, 2.74	0.63
RAI Stage [N (%)] ^1^					
Stage 0	9 (90)	7 (70)	0.453		
Stage I	1 (10)	2 (20)			0.346
Unknown	0	1 (10)			
Disease Duration (years)	5.0 ± 2.9	4.9 ± 2.7	0.937	−2.5, 2.7	0.04
Cytogenetics [N (%)] ^1^					
13q Deletion	7 (70)	5 (50)	0.361		0.20
17p Deletion	3 (30)	0	0.060		0.42
11q Deletion	2 (20)	0	0.136		0.33
Trisomy 12	0	2 (20)	0.136		0.33
TP53 Mutated	4 (40)	1 (10)	0.291		0.35
IGHV Mutated^1^	6 (60)	6 (60)	0.766		0.16
CD38 Expression >30% ^1^	1 (10)	0	0.119		0.46
WBC Counts (×10^3^/µL)	65 ± 54	51 ± 51	0.553	−35.1, 63.5	0.27
Lymphocytes	59 ± 52	44 ± 49	0.517	−32.7, 62.8	0.30
CD19+/CD5+ CLL-cells	48.1 ± 48.6	40.8 ± 41.2	0.359	−35.0, 49.7	0.16
CD4+ T-cells	1.6 ± 2.3	5.1 ± 7.4	0.169	−8.6, 1.6	0.64
CD8+ T-cells	0.8 ± 0.9	2.2 ± 2.3	0.088	−3.0, 0.2	0.80
Monocytes	1.4 ± 1.4	1.8 ± 2.3	0.694	−2.1, 1.5	0.21
Neutrophils	3.4 ± 1.6	4.9 ± 1.6	**0.046**	−3.0, −0.3	0.94
Neutrophil: T-cell	3.7 ± 5.7	2.4 ± 2.5	0.510	−2.2, 0.8	0.30
T-cell: Monocyte	2.1 ± 1.4	6.5 ± 7.6	0.086	−9.6, 0.7	0.81
Platelets (×10^3^/µL)	149 ± 43	196 ± 62	0.065	−96.4, 3.2	0.88
Hemoglobin (g/dL)	13.0 ± 1.7	13.6 ± 1.5	0.433	−2.1, 0.92	0.37
β2-microglobulin (mg/dL)	1.9 ± 0.7	2.4 ± 0.9	0.228	−1.3, 0.33	0.57
ICAM-1 (ng/mL)	709.1 ± 171.0	685.1 ± 158.6	0.755	−136, 184	0.15
sCD20 (pg/mL)	220.8 ± 306.9	118.2 ± 181.3	0.417	−158, 364	0.40
**Fitness and Performance**					
eVO_2peak_ (mL/kg/min)	34.2 ± 3.3	24.9 ± 3.2	**<0.001**	6.2, 12.4	2.86
6MWT (m)	500 ± 77	399 ± 107	**0.027**	12.6, 188	1.08
SPPB Score	12 ± 0	9.9 ± 2.4	**0.012**	0.52, 3.68	1.24
TUG (sec)	9.4 ± 1.6	11.6 ± 4.5	0.170	−5.6, 1.07	0.65
Grip Strength Right (kg)	33.3 ± 12.0	32.7 ± 16.7	0.930	−13.1, 14.3	0.04
Grip Strength Left (kg)	32.4 ± 14.3	30.5 ± 16.1	0.780	−12.4, 16.3	0.12
Best Grip (BMI normalized)	1.4 ± 0.4	1.1 ± 0.5	0.171	−0.15, 0.77	0.66
IPAQ (total hours/week)	31.9 ± 29.1	26.4 ± 16.8	0.610	−10.2, 50.6	0.23
SBAS [N (%)] ^1^					
Inactive	2 (20)	3 (30)	0.936		
Light	4 (40)	3 (30)			
Moderate	2 (20)	2 (20)			0.21
Hard	1 (10)	1 (10)			
Very Hard	1 (10)	1 (10)			

^1^ Chi-square mean ± SD. BMI (Body Mass Index); CLL-IPI (CLL-International Prognostic Index); IGHV (Immunoglobulin Heavy-Chain Gene); WBC (White Blood Cell); ICAM-1 (Intercellular Adhesion Molecule 1); sCD20 (soluble CD20); 6MWD (6-Minute Walk Test); SPPB (Short Physical Performance Battery); TUG (Timed-Up And Go); IPAQ (Incidental and Planned Activity Questionnaire); SBAS (Stanford Brief Activity Survey). Bolded values represent significant differences between groups. Continuous variables are mean ± SD, and nominal data is *N* (%).

**Table 2:** Select plasma NMR measurements of systemic inflammation, insulin resistance, lipids, and lipoproteins.

	CLL-FIT (*N* = 10)	CLL-UNFIT (*N* = 10)	*p*-Value	95% CI	Effect Size (*d*)
**Inflammation**					
GlycA (µmol/L)	354.7 ± 31.1	442.0 ± 97.0	**0.014**	−155.0, −19.6	1.21
**Insulin Resistance**					
LP-IR (1-100)	33.7 ± 18.1	65.7 ± 22.2	**0.007**	−54.3, −9.7	1.58
**Triglycerides**					
Total Triglycerides (mg/dL)	101.4 ± 40.6	165.2 ± 72.0	**0.025**	−118.7, 8.9	1.09
TRL Triglycerides (mg/dL)	78.1 ± 38.8	143.4 ± 67.7	**0.016**	−117.1, −13.5	1.18
Total TRLP (nmol/L)	127.6 ± 58.4	189.8 ± 70.8	**0.046**	−123.2, −1.2	0.96
Large (nmol/L)	2.5 ± 3.4	8.9 ± 6.2	**0.011**	−11.1, −1.7	1.28
Very Small (nmol/L)	73.4 ± 38.4	124.4 ± 63.7	**0.044**	−100.4, −1.6	0.97
**Cholesterol (mg/dL)**					
Total Cholesterol	194.4 ± 29.1	195.4 ± 39.4	0.949	−33.5, 31.5	0.03
TRL Cholesterol	22.5 ± 10.8	34.9 ± 13.3	**0.034**	−23.8, −1.0	1.02
LDL Cholesterol	106.3 ± 25.7	105.5 ± 29.7	0.949	−25.3, 26.9	0.03
HDL Cholesterol	65.5 ± 10.0	54.8 ± 11.6	**0.040**	0.5, 20.9	0.99
HDL-P (µmol/L)					
Total	24.7 ± 2.4	23.8 ± 4.2	0.559	−2.3, 4.1	0.26
Large	3.4 ± 1.4	1.9 ± 1.6	**0.043**	0.1, 2.9	1.00
**Lipoprotein Size (nm)**					
TRL	43.7 ± 4.7	50.3 ± 7.2	**0.027**	−12.3, −0.9	1.09
LDL	20.8 ± 0.4	20.4 ± 0.4	0.059	0.02, 0.8	1.00
HDL	9.2 ± 0.4	8.8 ± 0.5	0.073	−0.03, 0.8	0.88

LP-IR (Lipoprotein–Insulin Resistance); TRL (Triglyceride-Rich Lipoprotein); TRLP (Triglyceride-Rich Lipoprotein Particle); HDL (High-Density Lipoprotein); LDL (Low-Density Lipoprotein), BCAA (Branch-Chain Amino Acid). Bolded values represent significant differences between groups. Data are the mean ± SD.

## Data Availability

The datasets generated during the present study are not publicly available, owing to the risk of disclosure or deduction of private individual information, but they are available from the corresponding author upon reasonable request.

## Supplementary Materials

The following are available online at https://www.mdpi.com/article/10.3390/cells10051165/s1: Table S1: Complete list of miRNAs identified. Table S2: Normalized exosomal miRNA expression differences between CLL-FIT and CLL-UNFIT.
