# Supplementary material for: Physiological Fitness and the Pathophysiology of Chronic Lymphocytic Leukemia (CLL)
Source: Cells. 2021 May 11;10(5):1165. doi: 10.3390/cells10051165 (PMC8151485; doi:10.3390/cells10051165)
Supplement: Supplementary file 1 [file cells-10-01165-s001.zip › cells-1205200-supplementary.pdf]

Supplementary Table 1. Complete List of miRNAs identified.

miRNA	log2 Fold Change (CLL-FIT/CLL-UNFIT)	Log2 Fold (S.E.)	Wald Test Statistic	Wald Test *p* value
hsa-mir-101-2-3p	-0.983875786	0.36716	-2.679722209	0.0074
hsa-mir-199a-1-3p	0.88129529	0.33034	2.667857739	0.0076
hsa-mir-199a-2-3p	0.88129529	0.33034	2.667857739	0.0076
hsa-mir-199b-3p	0.88129529	0.33034	2.667857739	0.0076
hsa-mir-101-1-3p	-0.971037046	0.37353	-2.599613693	0.0093
hsa-mir-378a-3p	-2.282514775	0.90012	-2.535796826	0.0112
hsa-mir-32-5p	-2.470054298	0.98256	-2.513902987	0.0119
hsa-mir-24-1-3p	0.758239422	0.30543	2.482527222	0.013
hsa-mir-24-2-3p	0.758239422	0.30543	2.482527222	0.013
hsa-mir-130b-5p	2.702963742	1.09236	2.474433631	0.0133
hsa-mir-744-5p	0.966822005	0.39263	2.462433206	0.0138
hsa-mir-1301-3p	3.145610151	1.31455	2.392918164	0.0167
hsa-mir-328-3p	1.608130759	0.68509	2.347339234	0.0189
hsa-mir-4433b-3p	3.13815109	1.3475	2.328873325	0.0199
hsa-mir-4433b-5p	1.388521901	0.60249	2.304643691	0.0212
hsa-mir-383-5p	5.668882623	2.47465	2.29078449	0.022
hsa-mir-29c-3p	-1.069640841	0.47632	-2.245652119	0.0247
hsa-mir-16-1-5p	-0.727284521	0.33597	-2.164725857	0.0304
hsa-mir-16-2-5p	-0.727284521	0.33597	-2.164725857	0.0304
hsa-mir-151a-5p	0.97675396	0.45355	2.153560989	0.0313
hsa-let-7f-1-5p	-0.635424076	0.29586	-2.147697168	0.0317
hsa-mir-19b-1-3p	-0.998943501	0.46834	-2.132966674	0.0329
hsa-mir-19b-2-3p	-0.994104722	0.46627	-2.132056046	0.033
hsa-mir-183-5p	-1.020194315	0.48043	-2.123496223	0.0337
hsa-mir-451a	-0.885223579	0.42546	-2.080613285	0.0375
hsa-mir-324-3p	3.34659127	1.63312	2.049206459	0.0404
hsa-let-7f-2-5p	-0.590170504	0.2884	-2.046353137	0.0407
hsa-mir-151a-3p	0.582577816	0.28579	2.038501319	0.0415
hsa-mir-576-5p	-1.062177535	0.52628	-2.018270367	0.0436
hsa-mir-6772-3p	5.973170484	2.96149	2.016948525	0.0437
hsa-mir-182-5p	-0.837563539	0.4247	-1.97212436	0.0486
hsa-mir-1296-5p	3.726897256	1.89014	1.971759554	0.0486
hsa-mir-142-5p	-0.752124146	0.38777	-1.939613632	0.0524
hsa-mir-15a-5p	-0.71711871	0.37039	-1.936142158	0.0529
hsa-mir-96-5p	-1.314855247	0.68199	-1.927956332	0.0539
hsa-mir-1307-3p	0.837645679	0.44225	1.894057784	0.0582
hsa-let-7g-5p	-0.569977944	0.30771	-1.852294927	0.064
hsa-let-7a-1-5p	-0.531267809	0.29274	-1.814835503	0.0695
hsa-let-7a-2-5p	-0.531267809	0.29274	-1.814835503	0.0695
hsa-let-7a-3-5p	-0.531267809	0.29274	-1.814835503	0.0695
hsa-mir-339-5p	0.51066174	0.28264	1.806736469	0.0708
hsa-mir-199a-1-5p	1.975049998	1.09976	1.795891286	0.0725
hsa-mir-199a-2-5p	1.975049998	1.09976	1.795891286	0.0725
hsa-mir-191-5p	0.426561535	0.24535	1.738602443	0.0821
hsa-mir-29a-3p	-1.226522299	0.71389	-1.71808784	0.0858

hsa-mir-584-5p	0.606666997	0.35924	1.688734072	0.0913
hsa-mir-139-3p	0.936260741	0.55753	1.679300781	0.0931
hsa-mir-140-5p	1.24689876	0.75116	1.659954468	0.0969
hsa-mir-20b-5p	-0.934883701	0.56733	-1.647852597	0.0994
hsa-mir-223-3p	0.541384276	0.32938	1.643647946	0.1002
hsa-mir-3064-5p	3.89556136	2.38492	1.633417269	0.1024
hsa-mir-500a-3p	-1.126655578	0.7024	-1.604006715	0.1087
hsa-mir-10a-5p	-0.651213235	0.40674	-1.601036544	0.1094
hsa-mir-331-5p	4.73880117	2.96803	1.5966131	0.1104
hsa-mir-629-3p	-4.244348122	2.67072	-1.58921359	0.112
hsa-mir-664b-3p	-4.017289695	2.53324	-1.585833248	0.1128
hsa-mir-1291	-1.661940698	1.0522	-1.579492975	0.1142
hsa-mir-2355-3p	4.149533168	2.65535	1.562703966	0.1181
hsa-mir-6738-5p	4.463026457	2.97042	1.502491583	0.133
hsa-mir-133a-1-3p	2.136473363	1.42483	1.499454912	0.1338
hsa-mir-133a-2-3p	2.136473363	1.42483	1.499454912	0.1338
hsa-mir-142-3p	0.349845229	0.23339	1.498971514	0.1339
hsa-mir-483-5p	1.566411423	1.05549	1.484057072	0.1378
hsa-mir-5187-5p	1.805291799	1.21942	1.480456208	0.1388
hsa-mir-221-3p	0.492404648	0.33451	1.471998366	0.141
hsa-mir-143-3p	0.568444726	0.38817	1.464415718	0.1431
hsa-mir-30e-3p	-0.780048649	0.53283	-1.463969616	0.1432
hsa-mir-140-3p	-0.47168392	0.32297	-1.460475962	0.1442
hsa-mir-30a-5p	-0.592087769	0.40836	-1.449904436	0.1471
hsa-mir-181a-2-5p	-0.440722775	0.30479	-1.445981503	0.1482
hsa-mir-664b-5p	1.857625403	1.28671	1.443704221	0.1488
hsa-mir-660-5p	-0.731350959	0.50937	-1.435785567	0.1511
hsa-let-7g-3p	-4.240436069	2.95536	-1.434828286	0.1513
hsa-mir-505-5p	1.504533296	1.04949	1.433586627	0.1517
hsa-mir-1299	-2.015661936	1.41262	-1.42689401	0.1536
hsa-mir-320a	0.324779735	0.23536	1.379916189	0.1676
hsa-mir-4732-5p	-1.479082901	1.08592	-1.362057497	0.1732
hsa-mir-497-5p	-2.155441964	1.59736	-1.349377501	0.1772
hsa-mir-484	-0.603713321	0.44876	-1.345283012	0.1785
hsa-mir-93-5p	-0.35842289	0.26817	-1.336566345	0.1814
hsa-mir-3164	3.898097408	2.93199	1.329504889	0.1837
hsa-let-7b-5p	-0.487144465	0.36684	-1.327949971	0.1842
hsa-mir-651-5p	-2.649315881	1.99809	-1.325924204	0.1849
hsa-mir-144-3p	-0.70339845	0.53144	-1.323582713	0.1856
hsa-mir-27a-3p	-0.687787587	0.52055	-1.321266032	0.1864
hsa-mir-21-5p	-0.577539264	0.44548	-1.296437372	0.1948
hsa-mir-144-5p	-0.61077933	0.48262	-1.265560243	0.2057
hsa-mir-5010-3p	-3.74838597	2.96791	-1.262971665	0.2066
hsa-let-7c-5p	0.441837838	0.35343	1.25015088	0.2112
hsa-mir-3158-1-3p	-1.459726023	1.17272	-1.244738177	0.2132
hsa-mir-3158-2-3p	-1.459726023	1.17272	-1.244738177	0.2132
hsa-mir-1307-5p	1.200211251	0.96749	1.240547475	0.2148
hsa-mir-1287-5p	1.608184763	1.30476	1.232548282	0.2177
hsa-mir-376c-3p	-1.342712173	1.1127	-1.20671296	0.2275

hsa-mir-3200-3p	3.103176477	2.57273	1.206181625	0.2277
hsa-mir-181d-5p	1.99394513	1.66279	1.199156094	0.2305
hsa-mir-25-3p	-0.3641706	0.30582	-1.19081383	0.2337
hsa-mir-570-3p	-2.857004846	2.40372	-1.188576114	0.2346
hsa-mir-15b-3p	0.903633226	0.76148	1.186675883	0.2354
hsa-mir-362-5p	1.637914238	1.38069	1.186304002	0.2355
hsa-mir-31-5p	-2.703802932	2.27987	-1.185943668	0.2356
hsa-mir-30b-5p	-0.607985789	0.52084	-1.167324952	0.2431
hsa-mir-212-3p	3.463673702	2.98396	1.16076351	0.2457
hsa-mir-486-2-5p	-0.493572567	0.42601	-1.158604844	0.2466
hsa-mir-491-5p	0.81073414	0.70616	1.148089643	0.2509
hsa-let-7d-3p	1.001751966	0.87309	1.147369578	0.2512
hsa-mir-150-3p	-3.388448154	2.9711	-1.140470677	0.2541
hsa-mir-203a-3p	-2.667249535	2.3567	-1.131772687	0.2577
hsa-mir-6858-5p	1.584063222	1.40565	1.126928019	0.2598
hsa-mir-5698	3.343729312	2.97406	1.124296149	0.2609
hsa-mir-423-3p	0.336591286	0.2994	1.124202189	0.2609
hsa-mir-1294	-1.042776142	0.93021	-1.121016389	0.2623
hsa-mir-3064-3p	3.333949929	2.98636	1.116393602	0.2643
hsa-mir-3180-2	3.331709622	2.98639	1.115630111	0.2646
hsa-mir-3180-4	3.331709622	2.98639	1.115630111	0.2646
hsa-mir-3180-5	3.331709622	2.98639	1.115630111	0.2646
hsa-mir-92a-2-3p	-0.449038872	0.40295	-1.114385426	0.2651
hsa-mir-766-5p	3.323130745	2.98653	1.112706431	0.2658
hsa-let-7d-5p	-0.332395837	0.30114	-1.103797346	0.2697
hsa-mir-23a-3p	0.284144706	0.2588	1.09793917	0.2722
hsa-mir-190b	-1.968724688	1.79362	-1.097623767	0.2724
hsa-mir-335-3p	-1.029920973	0.93958	-1.096152013	0.273
hsa-mir-99b-3p	2.194749348	2.00433	1.095003674	0.2735
hsa-mir-486-1-5p	-0.429168897	0.39309	-1.091781631	0.2749
hsa-mir-130b-3p	-1.127433105	1.03524	-1.089052114	0.2761
hsa-mir-3200-5p	-3.094777686	2.84561	-1.0875607	0.2768
hsa-mir-361-3p	0.419272606	0.38642	1.085020186	0.2779
hsa-mir-7704	-3.197168331	2.97314	-1.075351171	0.2822
hsa-mir-7-1-5p	-0.438229113	0.40815	-1.073699792	0.283
hsa-mir-3144-3p	3.168918061	2.97653	1.064633578	0.287
hsa-mir-374b-5p	0.990212585	0.93098	1.06362797	0.2875
hsa-mir-224-5p	0.638471575	0.60309	1.058665143	0.2898
hsa-mir-585-3p	-3.141012384	2.97379	-1.056231652	0.2909
hsa-mir-128-2-3p	0.372999753	0.35386	1.054078666	0.2918
hsa-mir-629-5p	0.428326695	0.40673	1.053088705	0.2923
hsa-mir-340-5p	0.589314566	0.56054	1.051338137	0.2931
hsa-mir-3150b-3p	-3.125661517	2.97397	-1.051005013	0.2933
hsa-mir-4732-3p	-0.903155739	0.86624	-1.04261025	0.2971
hsa-mir-28-3p	0.370397656	0.3561	1.040152227	0.2983
hsa-mir-128-1-3p	0.353124571	0.34101	1.035521935	0.3004
hsa-mir-363-3p	-0.575093262	0.55593	-1.034476739	0.3009
hsa-mir-15b-5p	-0.30785793	0.29798	-1.033151978	0.3015
hsa-mir-181a-1-5p	-0.298280161	0.29018	-1.027924817	0.304

hsa-mir-92a-1-3p	-0.410013596	0.40209	-1.019708428	0.3079
hsa-mir-28-5p	-2.16503415	2.12925	-1.016806371	0.3092
hsa-mir-532-5p	-0.360212089	0.36018	-1.000086417	0.3173
hsa-mir-7-2-5p	-0.404392394	0.40799	-0.991174288	0.3216
hsa-mir-7-3-5p	-0.404392394	0.40799	-0.991174288	0.3216
hsa-mir-433-3p	2.941873896	2.99348	0.982759389	0.3257
hsa-mir-548ac	2.901867296	2.99432	0.969123648	0.3325
hsa-mir-196b-5p	-0.359039909	0.37849	-0.94860875	0.3428
hsa-mir-19a-3p	-0.557186371	0.59016	-0.9441336	0.3451
hsa-mir-1-1-3p	1.622975393	1.72304	0.941927949	0.3462
hsa-mir-1-2-3p	1.622975393	1.72304	0.941927949	0.3462
hsa-mir-939-5p	2.524382984	2.68058	0.941731891	0.3463
hsa-mir-548u	2.812373096	2.99628	0.938622394	0.3479
hsa-mir-185-3p	2.797473071	2.99661	0.933544498	0.3505
hsa-mir-424-5p	-1.249246606	1.38992	-0.898790946	0.3688
hsa-mir-107	-0.300912229	0.33522	-0.8976575	0.3694
hsa-mir-1288-3p	-2.656902516	2.98059	-0.891402027	0.3727
hsa-mir-320b-1	0.670591588	0.76095	0.881260057	0.3782
hsa-mir-320b-2	0.670591588	0.76095	0.881260057	0.3782
hsa-mir-3150a-5p	2.627042004	2.99993	0.875700119	0.3812
hsa-mir-30e-5p	-0.294007045	0.33702	-0.872376567	0.383
hsa-mir-206	1.569659152	1.79942	0.872315899	0.383
hsa-mir-324-5p	0.573213858	0.6609	0.867328367	0.3858
hsa-mir-509-1-3p	2.584370053	3.00063	0.861276983	0.3891
hsa-mir-509-2-3p	2.584370053	3.00063	0.861276983	0.3891
hsa-mir-509-3-3p	2.584370053	3.00063	0.861276983	0.3891
hsa-mir-26b-5p	-0.203933619	0.23689	-0.860871156	0.3893
hsa-mir-4746-5p	2.523122144	2.94426	0.856963108	0.3915
hsa-mir-122-5p	0.420232104	0.49261	0.853066388	0.3936
hsa-mir-222-3p	-0.446158492	0.5258	-0.848539811	0.3961
hsa-mir-3605-3p	1.535718265	1.81091	0.848038263	0.3964
hsa-mir-4747-5p	2.531115247	3.00152	0.843278364	0.3991
hsa-mir-636	2.531115247	3.00152	0.843278364	0.3991
hsa-mir-18a-5p	-0.655981756	0.78577	-0.834822631	0.4038
hsa-mir-326	-1.87474568	2.24649	-0.834522137	0.404
hsa-mir-7976	1.345429717	1.61339	0.833915221	0.4043
hsa-mir-1270	0.89190461	1.08908	0.818951324	0.4128
hsa-mir-106b-3p	-0.24391259	0.30189	-0.807948878	0.4191
hsa-mir-485-3p	0.960213735	1.19613	0.802767715	0.4221
hsa-mir-487a-3p	2.404471201	3.00377	0.800484584	0.4234
hsa-mir-1180-3p	-0.603673575	0.76323	-0.790944611	0.429
hsa-mir-345-5p	-0.618622023	0.78307	-0.789995676	0.4295
hsa-mir-516a-1-5p	2.346562509	3.00486	0.78092162	0.4348
hsa-mir-516a-2-5p	2.346562509	3.00486	0.78092162	0.4348
hsa-mir-423-5p	0.265452449	0.34008	0.780554926	0.4351
hsa-mir-654-3p	0.517190245	0.66347	0.779520054	0.4357
hsa-mir-7977	-2.323465293	2.98676	-0.777920908	0.4366
hsa-mir-146a-5p	0.292640558	0.37764	0.774918786	0.4384
hsa-mir-625-3p	-0.614544092	0.79599	-0.772048374	0.4401

hsa-mir-4661-5p	-2.298807596	2.98728	-0.769532633	0.4416
hsa-mir-483-3p	-2.298807596	2.98728	-0.769532633	0.4416
hsa-mir-29c-5p	-1.414377183	1.85754	-0.761426398	0.4464
hsa-mir-4326	-2.235956155	2.98863	-0.748154401	0.4544
hsa-mir-185-5p	-0.230168037	0.30844	-0.746243938	0.4555
hsa-mir-126-5p	-0.243199403	0.32662	-0.744602978	0.4565
hsa-mir-769-5p	-0.509823028	0.69264	-0.736058567	0.4617
hsa-mir-3681-5p	-2.193899418	2.98957	-0.733852009	0.463
hsa-mir-190a-5p	-0.352988191	0.48208	-0.732224631	0.464
hsa-mir-331-3p	1.051371835	1.43746	0.73141018	0.4645
hsa-mir-655-3p	-2.173515652	2.99003	-0.726920888	0.4673
hsa-mir-365a-3p	-1.306469462	1.80288	-0.724658675	0.4687
hsa-mir-365b-3p	-1.306469462	1.80288	-0.724658675	0.4687
hsa-mir-4446-3p	1.123634877	1.55339	0.723345334	0.4695
hsa-mir-93-3p	0.5497286	0.76406	0.719487353	0.4718
hsa-mir-154-5p	-1.02578571	1.42638	-0.719152417	0.472
hsa-mir-199b-5p	1.21151662	1.70902	0.708893628	0.4784
hsa-mir-873-5p	-2.097533195	2.96756	-0.706819739	0.4797
hsa-mir-99b-5p	0.246843379	0.34967	0.705937336	0.4802
hsa-mir-17-3p	-1.232787866	1.75814	-0.7011893	0.4832
hsa-mir-4678	-2.083543348	2.99216	-0.696334902	0.4862
hsa-mir-210-3p	0.865804118	1.25034	0.692457356	0.4887
hsa-mir-4742-5p	-2.071435596	2.99245	-0.692219883	0.4888
hsa-mir-34a-5p	-0.64929074	0.93959	-0.691039862	0.4895
hsa-mir-320c-1	0.426691848	0.6187	0.689659109	0.4904
hsa-mir-320c-2	0.426691848	0.6187	0.689659109	0.4904
hsa-mir-548j-3p	2.067843758	3.00938	0.68713178	0.492
hsa-mir-6758-5p	-2.036290668	2.99333	-0.680276751	0.4963
hsa-mir-10b-5p	-0.284500404	0.41887	-0.679207531	0.497
hsa-mir-181c-5p	-0.945489968	1.39344	-0.678529961	0.4974
hsa-mir-152-3p	0.439566519	0.6494	0.676884471	0.4985
hsa-mir-3127-3p	-2.02151234	2.9937	-0.675255373	0.4995
hsa-mir-5009-5p	-2.02151234	2.9937	-0.675255373	0.4995
hsa-mir-155-5p	-0.355229215	0.52646	-0.674756468	0.4998
hsa-mir-6503-5p	2.016471232	3.0103	0.669856871	0.5029
hsa-mir-1273h-3p	0.488230451	0.738	0.661554526	0.5083
hsa-mir-30d-5p	0.126095871	0.1911	0.659850732	0.5093
hsa-mir-501-3p	0.29483556	0.44858	0.657260419	0.511
hsa-mir-98-5p	-0.340355867	0.51904	-0.655746369	0.512
hsa-mir-184	1.046798261	1.60452	0.652404597	0.5141
hsa-mir-136-3p	-0.913442197	1.40313	-0.651004939	0.515
hsa-mir-598-3p	0.386072442	0.59872	0.644834766	0.519
hsa-mir-671-5p	-0.483400253	0.7523	-0.642565823	0.5205
hsa-mir-4742-3p	-1.883028381	2.95101	-0.638095667	0.5234
hsa-mir-503-5p	-1.010920657	1.60432	-0.630125956	0.5286
hsa-mir-590-3p	0.666525031	1.0589	0.629451142	0.5291
hsa-mir-21-3p	-1.343827722	2.14787	-0.625656698	0.5315
hsa-mir-342-3p	0.192250854	0.31149	0.61719589	0.5371
hsa-mir-126-3p	0.145621426	0.23645	0.615860607	0.538

hsa-mir-205-5p	0.485862517	0.79004	0.614981276	0.5386
hsa-mir-652-3p	-0.412189548	0.67076	-0.614514755	0.5389
hsa-mir-22-3p	-0.161893383	0.26391	-0.613435696	0.5396
hsa-mir-589-5p	-0.402217999	0.65774	-0.611516078	0.5409
hsa-mir-23b-3p	0.192870153	0.32039	0.601980524	0.5472
hsa-mir-27a-5p	0.418094296	0.69747	0.599446085	0.5489
hsa-mir-369-5p	0.609073747	1.02336	0.595171205	0.5517
hsa-mir-181b-1-5p	-0.389720091	0.65799	-0.592288056	0.5537
hsa-mir-329-1-3p	-0.739377762	1.27165	-0.581432077	0.5609
hsa-mir-329-2-3p	-0.739377762	1.27165	-0.581432077	0.5609
hsa-mir-221-5p	-1.724260885	2.96776	-0.580998008	0.5612
hsa-mir-148b-3p	0.173336744	0.30259	0.572845561	0.5667
hsa-let-7i-5p	-0.143058833	0.25126	-0.569357629	0.5691
hsa-mir-1306-5p	-0.79813189	1.40199	-0.569285436	0.5692
hsa-mir-664a-3p	1.087808509	1.91699	0.567455538	0.5704
hsa-mir-106b-5p	1.499065597	2.66243	0.563044657	0.5734
hsa-mir-196a-1-5p	1.408309876	2.53403	0.555758981	0.5784
hsa-mir-27b-3p	-0.195802202	0.35482	-0.55183852	0.5811
hsa-mir-1250-5p	-1.646299192	2.98377	-0.5517521	0.5811
hsa-mir-642a-3p	1.652291409	3.00404	0.55002376	0.5823
hsa-mir-486-1-3p	0.306589965	0.56971	0.538153083	0.5905
hsa-mir-486-2-3p	0.306589965	0.56971	0.538153083	0.5905
hsa-mir-411-5p	0.706681416	1.33458	0.529516604	0.5964
hsa-mir-424-3p	1.54737388	2.99936	0.515902149	0.6059
hsa-mir-194-2-5p	-0.333446997	0.64684	-0.515505336	0.6062
hsa-mir-181a-1-3p	0.38633716	0.75139	0.514164653	0.6071
hsa-mir-26a-1-5p	-0.140095512	0.27326	-0.512676456	0.6082
hsa-mir-26a-2-5p	-0.140095512	0.27326	-0.512676456	0.6082
hsa-mir-192-5p	-0.207821705	0.41295	-0.503256759	0.6148
hsa-mir-379-5p	-0.264283003	0.52524	-0.503162943	0.6148
hsa-mir-3130-1-3p	0.761605981	1.52356	0.49988525	0.6172
hsa-mir-3130-2-3p	0.761605981	1.52356	0.49988525	0.6172
hsa-mir-431-5p	-0.315319536	0.63121	-0.499545504	0.6174
hsa-mir-382-5p	-0.268838559	0.54371	-0.494453534	0.621
hsa-mir-218-1-5p	-0.812220469	1.64653	-0.493293533	0.6218
hsa-mir-378d-1	-0.731975264	1.49811	-0.488599454	0.6251
hsa-mir-378d-2	-0.731975264	1.49811	-0.488599454	0.6251
hsa-mir-9-1-5p	0.357021364	0.73602	0.485069267	0.6276
hsa-mir-9-2-5p	0.357021364	0.73602	0.485069267	0.6276
hsa-mir-9-3-5p	0.357021364	0.73602	0.485069267	0.6276
hsa-mir-454-3p	-0.22147426	0.45739	-0.484211756	0.6282
hsa-mir-339-3p	0.292705242	0.60595	0.483052342	0.6291
hsa-mir-124-1-5p	-1.410974375	3.00191	-0.470025274	0.6383
hsa-mir-124-2-5p	-1.410974375	3.00191	-0.470025274	0.6383
hsa-mir-124-3-5p	-1.410974375	3.00191	-0.470025274	0.6383
hsa-mir-139-5p	-0.268734967	0.57226	-0.469603394	0.6386
hsa-mir-194-1-5p	-0.30229033	0.64538	-0.468388695	0.6395
hsa-mir-4665-5p	-1.389997878	2.97746	-0.466839797	0.6406
hsa-mir-708-5p	0.861283474	1.84652	0.466435069	0.6409

hsa-mir-218-2-5p	-0.765308463	1.64318	-0.465749458	0.6414
hsa-mir-1260b	-0.946859248	2.04054	-0.464024449	0.6426
hsa-mir-425-5p	0.10800231	0.23479	0.459997189	0.6455
hsa-mir-323a-3p	-0.801786885	1.77038	-0.45289004	0.6506
hsa-mir-3176	-1.354686904	3.01108	-0.449901175	0.6528
hsa-mir-628-3p	0.631970062	1.42557	0.44330956	0.6575
hsa-mir-628-5p	-0.484288763	1.09945	-0.440482523	0.6596
hsa-mir-33a-5p	-1.238570987	2.81385	-0.440169221	0.6598
hsa-mir-9-1-3p	0.398150802	0.908	0.438493396	0.661
hsa-mir-9-2-3p	0.398150802	0.908	0.438493396	0.661
hsa-mir-9-3-3p	0.398150802	0.908	0.438493396	0.661
hsa-mir-296-5p	0.923528743	2.11213	0.437250535	0.6619
hsa-mir-877-5p	1.273536945	2.94289	0.432750631	0.6652
hsa-mir-92b-3p	-0.855936	1.98169	-0.431921362	0.6658
hsa-mir-92b-5p	1.193293258	2.83568	0.420814447	0.6739
hsa-mir-29b-1-3p	-0.167577243	0.40243	-0.41641227	0.6771
hsa-mir-29b-2-3p	-0.167577243	0.40243	-0.41641227	0.6771
hsa-mir-450b-5p	-1.236859535	2.97925	-0.415157401	0.678
hsa-mir-378a-5p	0.590634827	1.4317	0.412540322	0.6799
hsa-mir-1185-1-5p	1.194490473	2.96794	0.402464784	0.6873
hsa-mir-1185-2-5p	1.194490473	2.96794	0.402464784	0.6873
hsa-mir-454-5p	0.535702662	1.3531	0.39590723	0.6922
hsa-mir-487b-3p	0.37265413	0.94129	0.395899116	0.6922
hsa-mir-4723-5p	-1.188198733	3.0152	-0.394070179	0.6935
hsa-mir-204-5p	-0.535268441	1.37993	-0.387895053	0.6981
hsa-mir-150-5p	-0.150657505	0.39161	-0.384717218	0.7004
hsa-mir-1249-3p	1.133195781	2.99028	0.378959203	0.7047
hsa-mir-548k	-0.993771957	2.66054	-0.373522078	0.7088
hsa-mir-338-3p	0.226527881	0.60706	0.373156841	0.709
hsa-mir-1271-5p	-0.488341498	1.31853	-0.370367992	0.7111
hsa-let-7e-5p	0.143747211	0.38982	0.368751661	0.7123
hsa-mir-3920	-1.101310608	3.01714	-0.365018635	0.7151
hsa-mir-548e-5p	-0.819334678	2.25696	-0.363026007	0.7166
hsa-mir-941-1	-0.182209465	0.50333	-0.36201006	0.7173
hsa-mir-941-2	-0.182209465	0.50333	-0.36201006	0.7173
hsa-mir-409-5p	0.680943702	1.8978	0.358807202	0.7197
hsa-mir-3614-5p	0.634663711	1.78804	0.354949407	0.7226
hsa-mir-493-5p	0.450477454	1.29233	0.348578557	0.7274
hsa-mir-99a-5p	0.161244153	0.46346	0.347914996	0.7279
hsa-mir-135a-1-5p	-0.626130876	1.80199	-0.347467306	0.7282
hsa-mir-135a-2-5p	-0.626130876	1.80199	-0.347467306	0.7282
hsa-mir-330-5p	-0.51422637	1.53126	-0.335818277	0.737
hsa-mir-574-3p	0.232168375	0.69746	0.332877743	0.7392
hsa-mir-409-3p	-0.152042489	0.47164	-0.322369532	0.7472
hsa-mir-125a-5p	-0.141179584	0.44694	-0.315880942	0.7521
hsa-mir-2110	0.25926009	0.82784	0.31317781	0.7541
hsa-mir-3688-1-3p	-0.75527507	2.43184	-0.310577523	0.7561
hsa-mir-3688-2-3p	-0.75527507	2.43184	-0.310577523	0.7561
hsa-mir-550a-1-3p	-0.602502493	1.99701	-0.301702576	0.7629

hsa-mir-550a-2-3p	-0.602502493	1.99701	-0.301702576	0.7629
hsa-mir-550a-3-3p	-0.602502493	1.99701	-0.301702576	0.7629
hsa-mir-197-3p	0.153398128	0.51122	0.300061307	0.7641
hsa-mir-181b-2-5p	-0.182488695	0.60921	-0.29954761	0.7645
hsa-mir-4524a-3p	-0.878056149	2.95944	-0.296696678	0.7667
hsa-mir-889-3p	0.540575376	1.83882	0.293978754	0.7688
hsa-mir-148a-3p	-0.088458099	0.30816	-0.28705654	0.7741
hsa-mir-30d-3p	-0.623222512	2.19074	-0.284480133	0.776
hsa-mir-382-3p	0.558149491	1.96525	0.284009175	0.7764
hsa-mir-377-3p	-0.731130761	2.59242	-0.282026864	0.7779
hsa-mir-30a-3p	0.350269389	1.25738	0.278570012	0.7806
hsa-mir-335-5p	0.135560436	0.48855	0.277476201	0.7814
hsa-mir-125b-2-5p	0.137698835	0.49683	0.277153886	0.7817
hsa-mir-138-2-5p	-0.787970244	2.84755	-0.276718912	0.782
hsa-mir-141-3p	-0.23308995	0.84817	-0.274813807	0.7835
hsa-mir-6721-5p	-0.767800046	2.82084	-0.272188286	0.7855
hsa-mir-195-5p	-0.25774946	0.95325	-0.270390027	0.7869
hsa-mir-374a-5p	0.170063489	0.63203	0.269073312	0.7879
hsa-mir-197-5p	-0.542442856	2.07823	-0.261012213	0.7941
hsa-mir-3124-5p	0.76407435	2.94774	0.259206695	0.7955
hsa-mir-146b-5p	0.092016672	0.35672	0.257949713	0.7964
hsa-mir-3679-5p	-0.625557179	2.43197	-0.257222016	0.797
hsa-mir-874-5p	0.616859253	2.41218	0.255726693	0.7982
hsa-mir-1179	-0.49135462	1.94469	-0.252664165	0.8005
hsa-mir-124-1-3p	0.482188186	1.92368	0.250659414	0.8021
hsa-mir-124-2-3p	0.482188186	1.92368	0.250659414	0.8021
hsa-mir-124-3-3p	0.482188186	1.92368	0.250659414	0.8021
hsa-mir-130a-3p	-0.140026631	0.56045	-0.24984708	0.8027
hsa-mir-502-3p	-0.143480548	0.57639	-0.248931044	0.8034
hsa-mir-125b-1-5p	0.120015343	0.49182	0.244022767	0.8072
hsa-mir-196a-2-5p	-0.507267291	2.0795	-0.243937598	0.8073
hsa-mir-219a-2-5p	0.712629642	2.99412	0.238009665	0.8119
hsa-mir-671-3p	0.138520892	0.59965	0.231002129	0.8173
hsa-mir-320d-2	-0.158147063	0.68634	-0.230422028	0.8178
hsa-mir-337-5p	0.239735765	1.04949	0.228430508	0.8193
hsa-mir-1908-5p	0.487563816	2.13882	0.227959557	0.8197
hsa-mir-103a-1-3p	-0.047995252	0.21299	-0.225338028	0.8217
hsa-mir-103a-2-3p	-0.047995252	0.21299	-0.225338028	0.8217
hsa-mir-501-5p	-0.535132485	2.37864	-0.22497376	0.822
hsa-mir-191-3p	0.656897604	2.94962	0.222705971	0.8238
hsa-mir-17-5p	0.081462071	0.36693	0.22201034	0.8243
hsa-mir-361-5p	0.110486771	0.50887	0.217122356	0.8281
hsa-mir-145-5p	0.131275831	0.61236	0.214375798	0.8303
hsa-mir-942-5p	-0.186949401	0.87306	-0.214131821	0.8304
hsa-mir-3913-1-5p	0.275335027	1.31443	0.209470916	0.8341
hsa-mir-3913-2-5p	0.275335027	1.31443	0.209470916	0.8341
hsa-mir-425-3p	0.208508641	1.00766	0.206923005	0.8361
hsa-mir-219a-1-5p	-0.457785372	2.23384	-0.204932418	0.8376
hsa-mir-421	0.178352646	0.88011	0.202648718	0.8394

hsa-mir-625-5p	0.165197769	0.82364	0.200571072	0.841
hsa-mir-766-3p	0.161657598	0.8352	0.193555395	0.8465
hsa-mir-30c-1-5p	0.050705336	0.26416	0.191948431	0.8478
hsa-mir-30c-2-5p	0.050705336	0.26416	0.191948431	0.8478
hsa-mir-153-1-3p	0.475025002	2.49176	0.190638363	0.8488
hsa-mir-153-2-3p	0.475025002	2.49176	0.190638363	0.8488
hsa-mir-22-5p	-0.329576949	1.73193	-0.190294096	0.8491
hsa-mir-7706	0.445336595	2.34419	0.189974285	0.8493
hsa-mir-432-5p	0.0853604	0.46819	0.182319081	0.8553
hsa-mir-323b-3p	-0.124975916	0.69286	-0.180376258	0.8569
hsa-mir-1292-5p	0.324230503	1.80178	0.179949693	0.8572
hsa-mir-18a-3p	-0.209754771	1.2203	-0.171887362	0.8635
hsa-mir-149-5p	0.331182193	1.92726	0.17184062	0.8636
hsa-mir-769-3p	0.272051837	1.61029	0.168945583	0.8658
hsa-mir-374a-3p	-0.235114845	1.47087	-0.15984794	0.873
hsa-mir-488-3p	-0.450880825	2.83283	-0.159162599	0.8735
hsa-mir-4532	0.17537398	1.11159	0.157768925	0.8746
hsa-mir-193a-5p	-0.164043026	1.06618	-0.153860555	0.8777
hsa-mir-20a-5p	-0.053223071	0.37741	-0.14102141	0.8879
hsa-mir-1343-3p	0.360156224	2.64176	0.136331803	0.8916
hsa-mir-6842-3p	-0.242853852	1.94235	-0.125030626	0.9005
hsa-mir-548j-5p	0.061951991	0.53279	0.116279214	0.9074
hsa-mir-151b	-0.151239025	1.30267	-0.116099687	0.9076
hsa-mir-137	0.145735852	1.28179	0.113696964	0.9095
hsa-mir-186-5p	-0.031917712	0.28284	-0.112848629	0.9102
hsa-mir-381-3p	0.075254128	0.6834	0.11011795	0.9123
hsa-mir-4286	0.276581114	2.51994	0.109757068	0.9126
hsa-mir-664a-5p	-0.058254704	0.57138	-0.101955132	0.9188
hsa-mir-494-3p	0.120522326	1.22566	0.098332896	0.9217
hsa-mir-3074-5p	-0.283682045	2.9451	-0.096323523	0.9233
hsa-mir-340-3p	-0.269449636	2.95168	-0.091286846	0.9273
hsa-mir-874-3p	-0.094729951	1.04442	-0.090701114	0.9277
hsa-mir-138-1-5p	0.132987358	1.53486	0.086644731	0.931
hsa-mir-127-3p	0.047398045	0.56541	0.08382895	0.9332
hsa-mir-320d-1	-0.055253658	0.70456	-0.078422617	0.9375
hsa-mir-4772-3p	0.181243176	2.95363	0.061362949	0.9511
hsa-mir-4677-3p	0.104509155	1.74255	0.059974953	0.9522
hsa-let-7i-3p	-0.136698022	2.41953	-0.056497742	0.9549
hsa-mir-1224-5p	0.098866459	1.8148	0.054477913	0.9566
hsa-mir-369-3p	-0.099758769	1.9193	-0.051976646	0.9585
hsa-mir-100-5p	0.038706289	0.78553	0.049274107	0.9607
hsa-mir-25-5p	-0.052735013	1.09626	-0.048104398	0.9616
hsa-mir-532-3p	-0.045729343	0.96097	-0.047586466	0.962
hsa-mir-485-5p	0.060698119	1.3408	0.045269914	0.9639
hsa-mir-6852-5p	0.051623902	1.33638	0.038629677	0.9692
hsa-mir-362-3p	-0.089711666	2.33758	-0.038378036	0.9694
hsa-mir-132-3p	0.055712767	1.45477	0.038296696	0.9695
hsa-mir-493-3p	0.037401333	0.97916	0.038197267	0.9695
hsa-mir-3615	-0.015525649	0.45228	-0.03432759	0.9726

hsa-mir-223-5p	0.055189522	1.76859	0.031205293	0.9751
hsa-mir-200a-3p	0.065004144	2.14037	0.030370548	0.9758
hsa-mir-200c-3p	0.029119794	1.04211	0.027943242	0.9777
hsa-mir-548o-2-3p	-0.076854899	2.93694	-0.02616832	0.9791
hsa-mir-548o-3p	-0.076854899	2.93694	-0.02616832	0.9791
hsa-mir-885-5p	-0.076264255	3.00319	-0.025394391	0.9797
hsa-mir-301a-3p	-0.019982366	0.81673	-0.024466224	0.9805
hsa-mir-129-1-5p	-0.065146793	2.90104	-0.022456342	0.9821
hsa-mir-129-2-5p	-0.065146793	2.90104	-0.022456342	0.9821
hsa-mir-505-3p	0.019771952	1.02532	0.019283729	0.9846
hsa-mir-95-3p	-0.015299354	0.83069	-0.018417537	0.9853
hsa-mir-134-5p	-0.011557542	0.64621	-0.017885187	0.9857
hsa-mir-370-3p	-0.010172104	0.62974	-0.016152858	0.9871
hsa-mir-548a-1-3p	0.018906543	2.1648	0.008733612	0.993
hsa-mir-548a-2-3p	0.018906543	2.1648	0.008733612	0.993
hsa-mir-548a-3-3p	0.018906543	2.1648	0.008733612	0.993
hsa-mir-376a-1-3p	0.003327375	1.48629	0.00223871	0.9982
hsa-mir-376a-2-3p	0.003327375	1.48629	0.00223871	0.9982

Supplementary Table 2: Normalized exosomal miRNA expression differences between CLL-FIT and CLL-UNFIT

miRNA	CLL-Fit	CLL-Unfit	Log2 Fold Difference (SE)	Wald Test Statistic	Wald Test *p*-value
hsa-mir-101-2-3p	358.8 ± 110.7	709.1 ± 695.3	-0.98 (0.37)	-2.68	0.0074
hsa-mir-199a (1-3p and 2-3p)	2000.1 ± 806.3	1086.1 ± 597.8	0.88 (0.33)	2.67	0.0076
hsa-mir-199b-3p	2000.1 ± 806.3	1086.1 ± 597.8	0.88 (0.33)	2.67	0.0076
hsa-mir-101-1-3p	358.8 ± 110.7	702.8 ± 699.8	-0.97 (0.37)	-2.60	0.0093
hsa-mir-378a-3p	39.3 ± 31.4	192.0 ± 353.6	-2.28 (0.90)	-2.54	0.0112
hsa-mir-32-5p	6.7 ± 8.0	38.1 ± 51.7	-2.47 (0.98)	-2.51	0.0119
hsa-mir-24 (1-3p and 2-3p)	231.5 ± 67.5	137.2 ± 69.1	0.76 (0.31)	2.48	0.013
hsa-mir-130b-5p	18.9 ± 16.0	2.9 ± 5.1	2.70 (1.09)	2.47	0.0133
hsa-mir-744-5p	275.1 ± 148.2	141.2 ± 90.8	0.97 (0.39)	2.46	0.0138
hsa-mir-1301-3p	23.2 ± 25.9	2.6 ± 5.6	3.15 (1.31)	2.39	0.0167
hsa-mir-328-3p	69.0 ± 90.2	22.6 ± 20.4	1.61 (0.69)	2.35	0.0189
hsa-mir-4433b-3p	19.3 ± 22.1	2.1 ± 3.7	3.14 (1.35)	2.33	0.0199
hsa-mir-4433b-5p	109.4 ± 88.8	41.9 ± 30.5	1.39 (0.60)	2.30	0.0212
hsa-mir-383-5p	8.2 ± 24.7	0.0 ± 0.0	5.67 (2.47)	2.29	0.022
hsa-mir-29c-3p	895.7 ± 464.6	1879.8 ± 2799.6	-1.07 (0.48)	-2.25	0.0247
hsa-mir-16 (1-5p and 2-5p)	24287.9 ± 14781.3	40209.4 ± 18457.5	-0.73 (0.34)	-2.16	0.0304
hsa-mir-151a-5p	70.8 ± 39.3	35.6 ± 30.5	0.98 (0.45)	2.15	0.0313
hsa-let-7f-1-5p	3920.2 ± 1591.7	6090.8 ± 2622.7	-0.64 (0.30)	-2.15	0.0317
hsa-mir-19b (1-3p and 2-3p)	143.2 ± 83.7	286.4 ± 318.1	-1.00 (0.47)	-2.13	0.0329
hsa-mir-183-5p	76.8 ± 64.0	156.7 ± 136.5	-1.02 (0.48)	-2.12	0.0337
hsa-mir-451a	4683.4 ± 2758.3	8650.8 ± 4511.6	-0.89 (0.43)	-2.08	0.0375
hsa-mir-324-3p	7.7 ± 15.0	0.8 ± 1.2	3.35 (1.63)	2.05	0.0404
hsa-let-7f-2-5p	4069.2 ± 1621.0	6127.0 ± 2571.2	-0.59 (0.29)	-2.05	0.0407
hsa-mir-151a-3p	2250.9 ± 610.4	1503.6 ± 788.9	0.58 (0.29)	2.04	0.0415
hsa-mir-576-5p	31.0 ± 23.4	65.8 ± 44.6	-1.06 (0.53)	-2.02	0.0436
hsa-mir-6772-3p	10.0 ± 24.2	0.0 ± 0.0	5.97 (2.96)	2.02	0.0437
hsa-mir-182-5p	366.6 ± 229.6	655.2 ± 506.6	-0.84 (0.42)	-1.97	0.0486
hsa-mir-1296-5p	6.8 ± 9.1	0.4 ± 1.3	3.73 (1.89)	1.97	0.0486

Data are mean ± SD of normalized expression values, $\log_2$ fold change and SE for CLL-FIT/CLL-UNFIT.
